# Evidence‐based child and adolescent mental health care: The role of high‐quality and transparently reported evidence synthesis studies

**DOI:** 10.1002/jcv2.12197

**Published:** 2023-08-25

**Authors:** Alessio Bellato, Ioana Alina Cristea, Cinzia Del Giovane, Seena Fazel, Guilherme V. Polanczyk, Marco Solmi, Henrik Larsson

**Affiliations:** ^1^ School of Psychology University of Nottingham Malaysia Semenyih Malaysia; ^2^ Department of General Psychology University of Padova Padova Italy; ^3^ Department of Medical and Surgical Sciences for Children and Adults University‐Hospital of Modena and Reggio Emilia Modena Italy; ^4^ Institute of Primary Health Care (BIHAM) University of Bern Bern Switzerland; ^5^ Department of Psychiatry University of Oxford Oxford UK; ^6^ Department of Psychiatry Faculdade de Medicina FMUSP Universidade de São Paulo São Paulo Brazil; ^7^ Department of Psychiatry University of Ottawa Ottawa Ontario Canada; ^8^ Regional Centre for the Treatment of Eating Disorders and On Track: The Champlain First Episode Psychosis Program Department of Mental Health The Ottawa Hospital Ottawa Ontario Canada; ^9^ Ottawa Hospital Research Institute (OHRI) Clinical Epidemiology Program University of Ottawa Ottawa Ontario Canada; ^10^ Department of Child and Adolescent Psychiatry Charité Universitätsmedizin Berlin Germany; ^11^ School of Medical Sciences Örebro University Örebro Sweden

**Keywords:** evidence‐based practice, mental health, meta‐analysis, systematic review

The publication of evidence synthesis studies (e.g., systematic reviews, meta‐analyses of aggregated data or individual participant data, network meta‐analyses, umbrella reviews) has grown exponentially in recent decades, with many placing these studies at the top of the pyramid of what is considered *good* evidence (Murad et al., 2016). Evidence synthesis studies integrate and analyse the collective evidence from multiple sources, thus providing comprehensive overviews and analyses of the available literature. Importantly, clinicians, policymakers and researchers make informed decisions, suggest healthcare policies, and guide clinical practice, based on such studies. It is therefore important to ensure that high‐quality studies are conducted and published according to specific standardised protocols, to make sure that the evidence synthesis remains rigorous, accessible, and informative. The 13 evidence synthesis studies published in the current special issue of *JCPP Advances* report comprehensive overviews of several important areas in child and adolescent mental health.

An important focus of the studies in the special issue is on outcomes and prognosis, such as those demonstrating an association between Attention‐Deficit/Hyperactivity Disorder (ADHD) and cardiovascular problems (Li et al., 2023) and sleep problems (Marten et al., 2023), as well as for poor health‐related quality of life associated with low socio‐economic status amongst children and adolescents with ADHD (Sevastidis et al., 2023). Bogdan et al. (2023) presented a comprehensive summary of the main characteristics of longitudinal studies investigating child and adolescent mental health conditions in the general population; Aymerich et al. (2023) found that internalising and externalising problems are present in children with enuresis or encopresis; while Pollard et al. (2023) observed that anxiety problems during childhood are associated with multifaceted poor outcomes and considerable economic costs.

Another key focus was on early predictors, including one study reporting an association between markers of autonomic functioning and self‐injurious thoughts and behaviours in children and young people (Bellato et al., 2023), and another showing that sleep disturbances are transdiagnostic mediating factors of the relationship between adverse childhood experiences and psychopathology in children and adolescents (Liu et al., 2023).

Other studies in the current issue focused on interventions. For example, studies reported evidence for the effectiveness of stimulant medication for pre‐schoolers with ADHD (Sugaya et al., 2023), and long‐term benefits of behavioural parent training for children with ADHD (Doffer, in press). Keiller et al. (2023) found preliminary evidence of the effectiveness of dramatherapy for reducing emotional distress in children and young people, but suggested more methodologically rigorous studies are needed. Similarly, Hipolito et al. (2023) highlighted the lack of clear evidence about the effectiveness of non‐pharmacological interventions (e.g., behavioural therapy) for children and young people with selective mutism. Lastly, Cawthorne et al. (2023) investigated whether the modest efficacy of cognitive‐behavioural therapy for adolescents with anxiety disorders could be explained by the lack of randomised controlled trials (RCTs) conducted in this population; they found that in most cases single‐case experimental designs were not followed up with a RCT, highlighting an important gap that future research should address.

These papers not only focused on important research questions but also showcased recent developments in methodology for evidence synthesis and good practices for reporting findings of systematic reviews and meta‐analyses. In particular, Liu et al. (2023) used meta‐analytic structural equation modelling as their primary analytic method. This novel methodology for evidence synthesis allows to combine the strengths of meta‐analysis and structural equation modelling for investigating complex relationships between different outcome measures (in this case, adverse childhood experiences, sleep problems, and psychopathology). We would also like to commend Sugaya et al. (2023) for concluding their paper with a “Practical guidance: clinical recommendations” section. This should be more commonly done, since it provides clinical professionals with a brief and thorough summary of the evidence about a clinically relevant topic, and a clear set of recommendations for clinical practice.

One of the goals of this special issue was to publish high‐quality evidence synthesis studies that could provide guidance for future research, both in the short‐ and the long‐term. A protocol template was included in the “Call for Papers” for this special issue, and authors completed it before they were invited by the editors to submit the final paper. This approach probably encouraged authors to plan and structure their studies based on certain guidelines and criteria, which however are not standardly adopted across journals. Consensus shall be sought across evidence synthesis experts to identify and agree upon good practices that authors can follow when planning, conducting, and reporting evidence synthesis studies. Moreover, to reduce inconsistency in relation to quality appraisal of systematic reviews and/or meta‐analyses, we think it would be important to provide peer‐reviewers with specific editorial guidelines in relation to what criteria to consider when commenting on the quality of manuscript reporting evidence synthesis data (Gates et al., 2020); we aim to do this in the future for *JCPP Advances*.

We experimentally appraised the quality of the papers published in the current special issue to evaluate the overall quality of the reports and how much open science practices were followed. Among the instruments commonly used for evaluating the quality of systematic reviews and meta‐analyses, we used AMSTAR‐2 (Shea et al., 2017). All 13 studies were consistent in reporting their research questions based on the components of PICO and following PRISMA guidelines, they all included a protocol that was published before conducting the study (generally, in PROSPERO or OSF), used a comprehensive search strategy (at least in four separate online databases), reported detailed information about the studies included in the systematic review, reported any potential conflicts of interest and main funding sources, and used an appropriate instrument to assess risk of bias/study quality (see Figure 1). However, not all studies conducted independent screening (i.e., more than one author independently checking each title/abstract/full‐text), which—however—for this screening stage can be reasonably done for a proportion (e.g., 20% of included studies), or data extraction (or they did not report having done so). Considering the recent advancements in Artificial Intelligence (AI) technology, and its potential use for screening articles in systematic reviews (van Dijk et al., 2023), it is important that future evidence synthesis studies report information about the screening process transparently.

**FIGURE 1:**
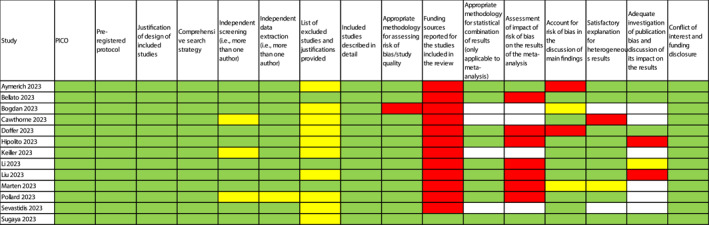
Summary of AMSTAR‐2 items scored for each study included in the current *JCPP Advances* special issue (green: YES; yellow: Probably yes; red: NO; white: not applicable).

Eight studies (out of 13) included a meta‐analysis. Although risk of bias and publication bias were generally assessed accurately (i.e., by using appropriate statistical tests and reporting information precisely), they were often not considered as potentially confounding elements in the analyses. For example, in only two studies (out of eight), sensitivity analyses or meta‐regressions were used to assess if and how much the inclusion of low‐quality studies or highly biased studies affected the main findings of the meta‐analysis.

Five studies provided a link to an external repository where raw data and analysis code had been stored, and two reported that data were available upon request. Making data and codes publicly available is particularly important, not only because following open science practices is a central component of *JCPP Advances*, but also because increased adherence to such principals is likely to improve transparency in disseminating evidence‐based findings and facilitate further collaborations. For example, secondary analyses or larger meta‐analyses (e.g., umbrella reviews and network meta‐analyses) could be conducted easily if data from individual studies are publicly available. However, we also acknowledge that in some cases sharing data publicly may not be possible; thus, reporting the main findings transparently (e.g., by providing forest plots, codes and outputs) is crucial.

We would also like to highlight that no umbrella review was submitted for this special issue: umbrella reviews are powerful tools to appraise evidence from multiple meta‐analyses (see, for example, Arrondo et al., 2022), hence we encourage authors to submit this type of evidence synthesis studies to *JCPP Advances*.

## CONCLUSIONS AND FUTURE DIRECTIONS

Considering the focus of this special issue and interest of *JCPP Advances* in publishing high‐quality evidence synthesis studies within the field of child and adolescent mental health, we would like to suggest good practices that we encourage research teams to follow when preparing evidence synthesis studies for submission to this journal. This list will be also helpful for reviewers, who will be encouraged to use it as a guideline when appraising the suitability of evidence synthesis papers for *JCPP Advances*.

- Report the main research question in PICO/PECO format, for example, in a separate table.
- Besides reporting detailed information about the studies that were included in the systematic review, also include (e.g., in appendix) a full list of articles that were excluded at full‐text screening (if possible, with reasons for exclusions, although this may not be necessary or feasible in larger studies, for which a clear PRISMA flowchart may suffice).
- Report detailed information about the screening process, including a description of how many authors completed this task, and if this was done independently. If an AI‐assisted software was used at any stage of the process, this shall be acknowledged.
- Consider how much risk of bias/study quality, publication bias, or heterogeneity, might have confounded the results of the meta analysis and, if appropriate, conduct secondary analyses to control for potential sources of bias. For example, include an assessment of the confidence of the estimates, by using the GRADE system.
- In line with open science practices, make codes and outputs publicly available (and consider making data available) to improve transparency, facilitate reproducibility, and promote further collaborations and advancements in evidence synthesis practice.
- Include a lay summary to present the main findings of the study and potential implications or recommendations for clinical practice.

## AUTHOR CONTRIBUTIONS

**Alessio Bellato:** Conceptualization; visualization; writing – original draft. **Ioana Alina Cristea, Cinzia Del Giovane, Seena Fazel, Guilherme V. Polanczyk, Marco Solmi:** Writing – review and editing. **Henrik Larsson:** Conceptualization; supervision; writing – original draft.

## CONFLICT OF INTEREST STATEMENT

Alessio Bellato, Henrik Larsson, Guilherme V. Polanczyk and Marco Solmi are authors of some of the papers included in the current issue and discussed in this editorial. Henrik Larsson, is Editor in Chief of JCPP Advances. Guilherme V. Polanczyk and Marco Solmi are Joint Editors for JCPP Advances. Seena Fazel serves on the JCPP Advances Editorial Advisory Board. Henrik Larsson reports receiving grants from Shire Pharmaceuticals; personal fees from and serving as a speaker for Medice, Shire/Takeda Pharmaceuticals and Evolan Pharma AB; all outside the submitted work. Marco Solmi received honoraria and has been a consultant for AbbVie, Angelini, Lundbeck, Otsuka. In the past 3 years, Guilherme V. Polanczyk has been consultant, advisory board member, and/or speaker for Aché, Abbott, Apsen, Medice, Novo Nordisk and Takeda, and received royalties from Editora Manole. The remaining authors have declared that they have no competing or potential conflicts of interest.

## Data Availability

NA.
